# Sub-inhibitory concentration of biogenic selenium nanoparticles lacks post antifungal effect for *Aspergillus niger* and *Candida albicans* and stimulates the growth of *Aspergillus niger*

**Published:** 2013-03

**Authors:** Zahra Bahri Kazempour, Mohammad Hossein Yazdi, Fatemeh Rafii, Ahmad Reza Shahverdi

**Affiliations:** 1 Department of Pharmaceutical Biotechnology and Biotechnology Research Centre, School of Pharmacy, Tehran University of Medical Sciences, Tehran, Iran; 2 Division of Microbiology National Center for Toxicological Research, Jefferson, AR 72079, U.S.A

**Keywords:** Selenium nanoparticle, *Aspergillus niger*, *Candida albicans*, Antifungal activity

## Abstract

**Background:**

The antifungal activity of selenium nanoparticles (Se NPs) prepared by *Klebsiella pneumoniae* has been reported previously for different fungi. In the present study, freshly prepared Se NPs produced by *K. pneumoniae* were purified and characterized by transmission electron microscopy and Energy-Dispersive X-ray spectroscopy (EDS) and its post antifungal effects for two fungi were evaluated.

**Materials and Methods:**

The minimum inhibitory concentrations (MICs) of Se NPs, determined by serial dilution were 250 µg/ml for *Aspergillus niger* and 2,000 µg/ml for *Candida albicans*. The effect of exposure of *A. niger* and *C. albicans* to Se NPs on later growth was evaluated by incubating the fungi for 1 hour at 25 °C in media containing 0, 1, 2 and 4 x MIC of Se NPs and diluting the cultures 100 times with Se free medium. The kinetics of growth of the fungi in control cultures and in non-toxic Se NPs concentration of, 0.01 × MIC, 0.02 × MIC or 0.04 × MIC were measured.

**Results:**

The exposure of *A. niger* and *C. albicans* to 2 and 4 x MIC of Se NPs stimulated the growth of both fungi in the absence of toxic concentrations of Se. The strongest stimulation was observed for *A. niger*.

**Conclusion:**

It is concluded that exposure to high concentration of the Se NPs did not have any post-inhibitory effect on *A. niger* and *C. albicans* and that trace amounts of this element promoted growth of both fungi in a dose- dependent-manner. The role of nanoparticles serving as needed trace elements and development of microorganism tolerance to nanoparticles should not be dismissed while considering therapeutic potential.

## Introduction

Nanotechnology allows the synthesis of nanoparticles (NPs) with novel antimicrobial properties (1–4). There is an increasing interest in the use of metallic or non-metallic NPs in health products for their antibiotic properties (5–9). The antifungal activity of silver NPs has been studied extensively (9–12). We have recently reported the synthesis and antifungal activity of biogenic selenium nanoparticles (Se NPs) prepared by *Klebsiella pneumoniae* (13, 14). Currently, high-strength selenium disulfide (2.5%) is available by prescription for the treatment of dandruff and seborrheic dermatitis (15). In addition, selenium disulfide is commercially available in a concentration of 1% as an over the counter antifungal agent in shampoos for the treatment of dandruff and seborrheic dermatitis. The toxicity reported for elemental selenium (Se^0^) is lower than the toxicity of selenate (Se^+2^) or selenite (Se^+4^) ions (16) and this element as nano-particles may be a candidate for replacement of other forms of selenium in the future. Post-antibiotic effect (PAE) defines the potential of a substance to delay re-growth of a microbial population after short-term exposure and removal of an antimicrobial compound (17, 18). Determination of the PAE is recommended in further evaluation of all new antimicrobial agents because it influences optimal antimicrobial dosing intervals. In this investigation, we have evaluated the effect of pre-exposure to biogenic Se NPs on the growth of test strains based on the method published by Jacobs *et al*. (17).

## MATERIALS AND METHODS

### Biosynthesis of Se NPs

Biogenic Se NPs were prepared using a recently described method (13). Briefly, cultures of *K. pneumoniae* were grown in tryptic soy broth (TSB) to an OD_600_ of 1.0 at 37°C. Fresh TSB at pH 7.2 was supplemented with 200 mg/l Se^+4^ (equal to 559.19 mg of selenium chloride) and inoculated with 1% (v/v) of a bacterial culture. The culture flasks were incubated at 37°C for 24 h. *K. pneumoniae* cells containing red selenium particles were disrupted by autoclaving at 121°C, 1.2 kg/cm^2^ for 20 min. The released Se NPs were centrifuged at 25,000 x g for 15 min and washed three times with distilled water. The washed samples were sonicated for 10 min (Techno-Gaz, Tecna6) and the physical and chemical properties of the samples were confirmed by transmission electron microscopy (TEM) and energy-dispersive X-ray spectroscopy (EDS).

### Biological assay

The antifungal activity of the biogenic Se NPs was determined for *Aspergillus niger* TUMS R124 and *Candida albicans* TUMS R152, which were kindly provided by Dr. Sassan Rezaie from the Culture Collection of the Department of Medical Mycology and Parasitology, School of Public Health, Tehran University of Medical Sciences, Tehran, Iran. The strains grew in Sabouraud dextrose broth (SDB) at 25°C. These conditions support the growth of a variety of fungi and are useful for the growth of fungi associated with skin. A conventional serial dilution method was used to test the susceptibility of *A. niger* and *C. albicans* to Se NPs (19) with an inoculum of approximately 10^4^ colony-forming units (CFU)/ml. The SDB culture media were supplemented with serial two-fold dilution of biogenic Se NPs, ranging from 62.5 to 8000 µg/ml. The minimum concentrations of Se NPs capable of inhibiting visible growth of the fungal strains during 48 h of incubation at 25°C were determined and reported as MICs. All experiments were repeated three times.

The effect of pre-exposure of the fungi to lethal concentrations of Se NPs (MIC, 2 × MIC and 4 × MIC) on the growth of test strains were determined based on the method published by Jacobs et al (17) as follows. Both of the test strains were exposed to Se NPs concentrations of (MIC, 2 × MIC and 4 × MIC) in SDB medium. Control cultures for each test strains were also prepared in SDB without Se NPs. After incubation for one hour at 25°C in a shaking incubator (150 rpm), all cultures were diluted with pre-warmed SDB at a ratio of 1/100 and incubated at 25°C for additional 48 h. The kinetics of growth was monitored by measuring the turbidity at OD_600_ at different intervals during incubation. Sterile SDB media containing Se NPs at concentrations of (0.01 MIC, 0.02 × MIC, and 0.04 × MIC) was used as reference controls. All samples were homogenized before measuring optical densities.

## RESULTS AND DISCUSSION

**Fig. 1:**
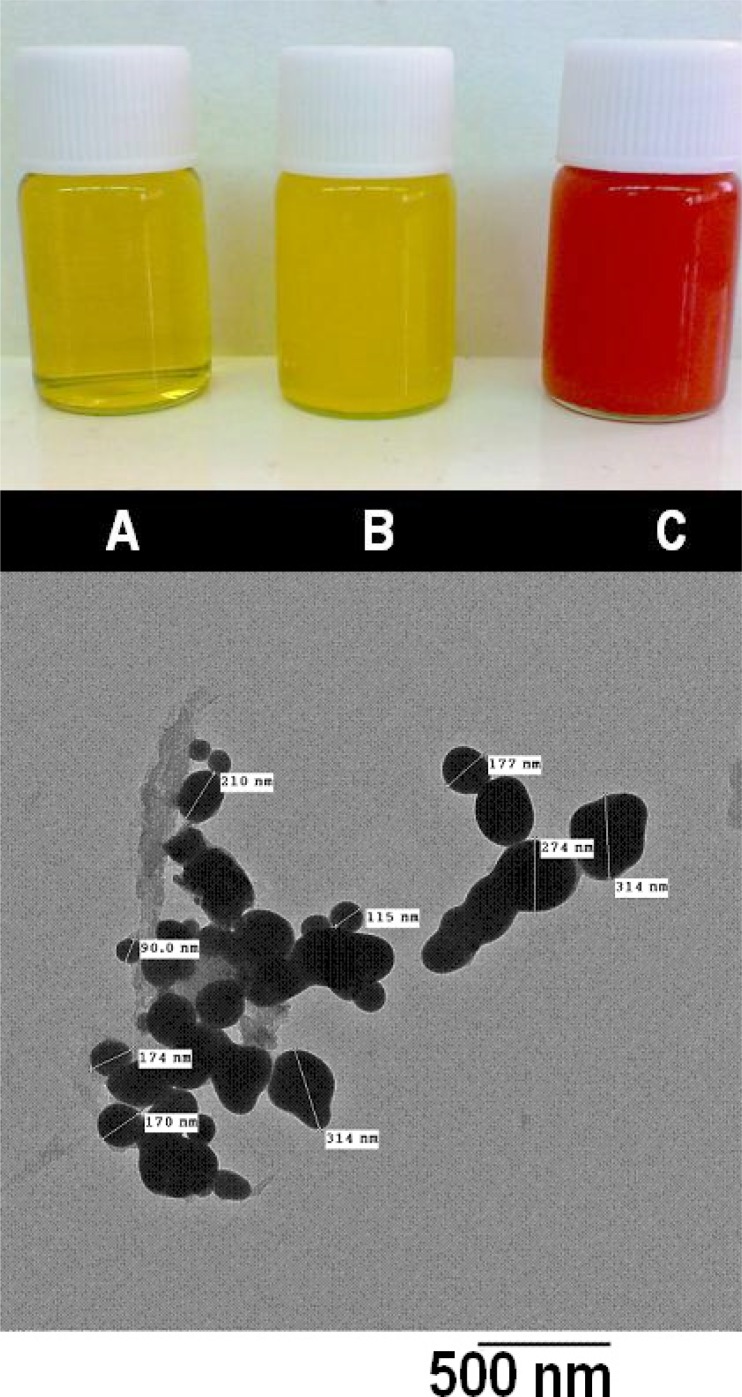
Upper panel (a) is an illustration of tubes containing tryptic soy broth before inoculation (tube A) and after inoculation with *K. pneumoniae* in the absence of Se^4+^ ion (tube B) and presence of Se^4+^ ions (tube C). The lower panel (b) represents a TEM micrograph of washed biogenic Se NPs obtained from *K. pneumoniae* cultures.

**Fig. 2:**
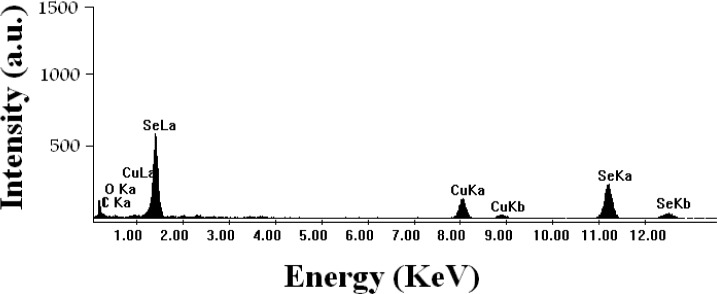
EDS spectrum of purified Se NPs. Selenium X-ray emission peaks are labeled. Strong signals from the atoms in the NPs, observed in the spectrum confirm the reduction of selenium ions to selenium metalloid NPs.

In the present investigation, biogenic Se NPs (ranging in size 90- 320 nm) were prepared by *K. pneumoniae* and their antifungal activities against *A. niger* and *C. albicans* were studied. After the incubation of *K. pneumoniae* with Se^+4^ the culture developed a red color. The Se NPs were released during autoclaving. Fig. 1a (top panel) illustrates the differences in the appearance of TSB medium before and after inoculation with *K. pneumoniae* and incubation for 24 h with and without Se^+4^ ions. The presence and the size of NPs were verified by the TEM micrograph of released Se NPs (Fig. 1b, bottom panel). The EDS analysis of the biogenic Se NPs confirmed the presence of elemental Se NPs signals (Fig. 2). The selenium nanocrystallites displayed optical absorption bands with peaks at 1.5, 11.2, and 12.5 keV, which is typical of metallic selenium nanocrystallites (18).

**Fig. 3:**
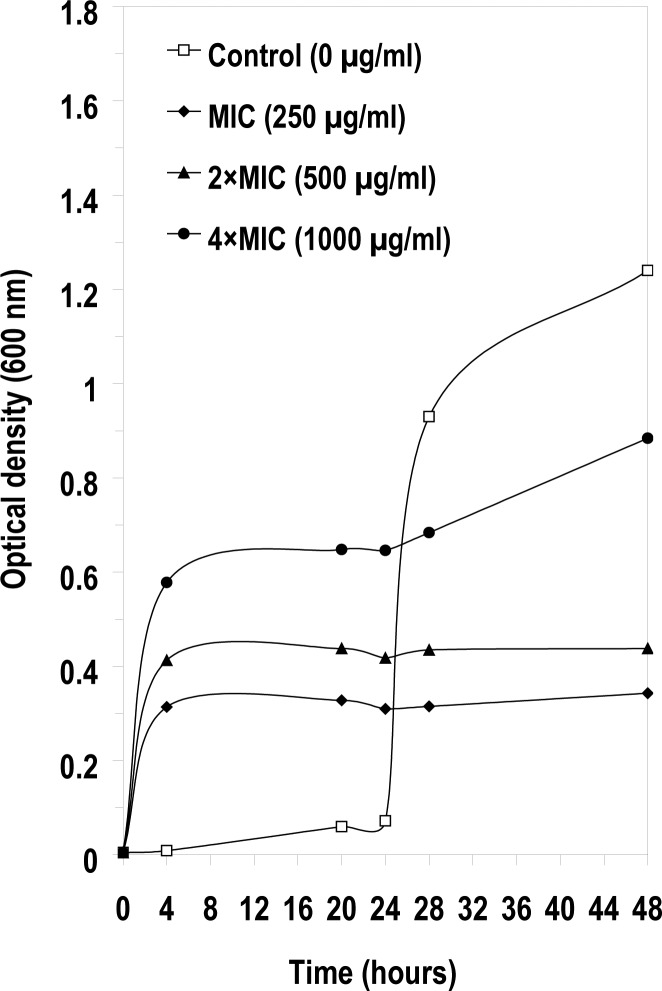
The post antifungal effect of Se NPs on the growth of *Aspergillus niger*.

**Fig. 4:**
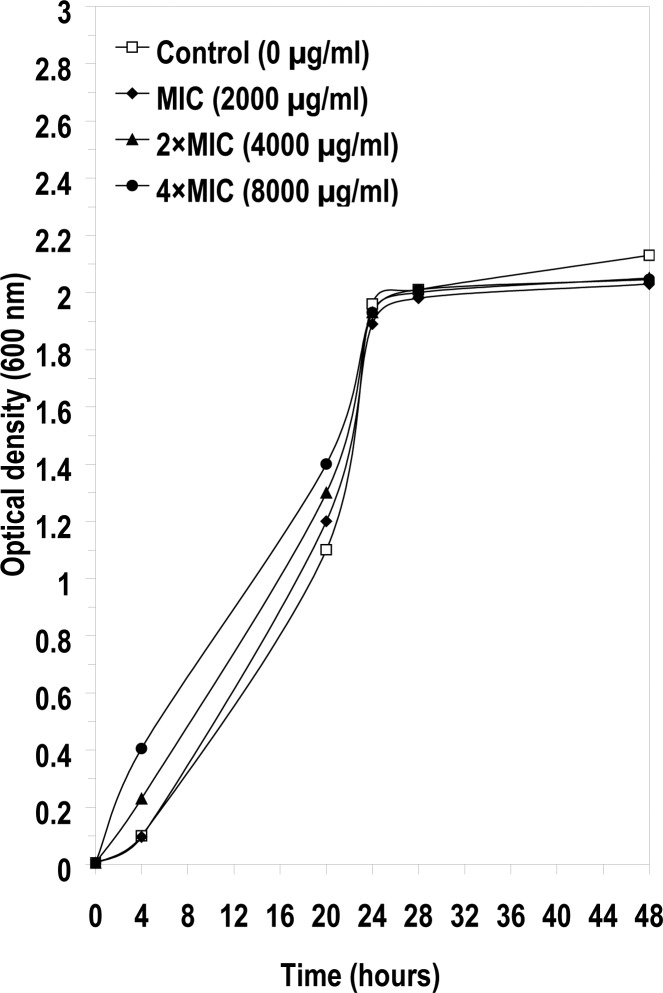
The post antifungal effect of Se NPs on the growth of *Candida albicans*.

The MIC values of Se NPs were 250 µg/ml for *A. niger* and 2000 µg/ml for *C. albicans*. The effects of pre-exposure of *A. niger* and *C. albicans* to high concentrations of NPs (MIC, 2 × MIC and 4 × MIC) on the growth of these strains in presence of trace amounts of the NPs (0.01 x MIC, 0.02 × MIC and 0.04 × MIC) were also evaluated (Figs. 3 and 4). Contrary to the post- antibiotic effect observed in bacteria on the re-growth of bacteria in the sub-inhibitory antibiotic concentration (17, 20), no significant differences in growth of *C. albicans* were observed in the presence or absence of Se NPs during growth in sub-inhibitory concentrations (20-80 µg/ml) of Se NPs (Fig. 4). However, there were differences in the growth of *A. niger* in lower subinhibitory concentrations (2.5-10 µg/ml) of Se NPs (Fig. 3). Surprisingly, there was higher growth in cultures containing 10 µg/ml of Se NPs (0.04 × MIC) than at 2.5µg/ml (0.01 × MIC). In addition a much longer lag phase was observed in the growth of *A. niger* in the medium without any NPs; however, after 24 h a more rapid growth and higher cell OD were observed in medium without NPs (Fig. 3). Selenium did not exhibit a PAE effect at the tested concentrations against these two fungal test strains, but showed considerable stimulation effect on the growth of *A. niger* in early stages of growth.

The results demonstrate that the pre-exposure of *A. nig*er to 1-4 times MIC of Se NPs resulted in the decrease in the lag phase of this strain during growth with sub-inhibitory concentrations of NPs, in comparison with the growth in medium without Se NPs by *A. niger* (Fig. 3). It is possible that the growth of *A. niger* may have been stimulated in the presence of selenium, because of its physiological effect on particular enzyme(s) in the cell. Abbass and Razak (1991) showed that *Aspergillus terreus* synthesized selenium-binding protein in the medium that was amended with 0.1% sodium selenite (20). Similarly Kulys et al. (1993) observed high electron transfer rates of glucose oxidase catalyzed oxidation of glucose in *A. niger* in the presence of selenium (21). It appears that *C. albicans* has more tolerance than *A. niger* for this compound since the MIC of Se NPs for *A. niger* was almost 10 times lower than the MIC for *C. albicans*.

In addition to their level of tolerance to Se NPs the growth rate of *A. niger* and *C. albicans* were different as evident from the slope of the growth of these fungi (Figs. 3 and 4).

Although initially there was a delay in the growth of control *A. niger*, after 48 hours it grow substantially more than other strains in sub-inhibitory concentrations of Se NPs. We do not know if the unknown physiological changes have occurred in this fungus during pre-exposed to Se NPs which may also have effected its growth including germination of its spores. This is the first study in which the PAE of Se NPs for different fungal strains has been investigated. Although the antifungal activity of nanoparticles against different fungal strains has been reported (5, 14), the PAE has not been measured.

In PAE assay, Se NPs not only did not exhibit an inhibitory effect at the tested concentrations against selected fungal test strains, but also showed considerable stimulation of the growth of one of the test strains possibly serving as trace elements in yet unknown metabolic activity. This may indicate that limited exposure of *A. niger* or *C. albicans* to Se NPs during medication of some dermatitis caused by these fungi may be harmful and may prompt the re-growth of these opportunistic pathogens on the surface of the skin.

## References

[1] Gajjar P, Pettee B, Britt D W, Huang W, Johnson W P, Anderson A J (2009). Antimicrobial activities of commercial nanoparticles against an environmental soil microbe, *Pseudomonas putida* KT2440. J Biol Eng.

[2] Liu L, Xu K, Wang H, Tan PK J, Fan W, Venkatraman SS (2009). Self-assembled cationic peptide nanoparticles as an efficient antimicrobial agent. Nat Nanotechnol.

[3] Ren G, Hu D, Cheng EW, Vargas-Reus MA, Reip P, Allaker RP (2009). Characterisation of copper oxide nanoparticles for antimicrobial applications. Int J Antimicrobial agents.

[4] Sadiq IM, Chowdhury B, Chandrasekaran N, Mukherjee A (2009). Antimicrobial sensitivity of Escherichia coli to alumina nanoparticles. Nanomedicine.

[5] Chladek G, Mertas A, Barszczewska-Rybarek I, Nalewajek T, Zmudzki J, Król W (2011). Antifungal activity of denture soft lining material modified by silver nanoparticles-a pilot study. Int J Mol Sci.

[6] Esteban-Tejeda L, Malpartida F, Esteban-Cubillo A, Pecharromán C, Moya JS (2009). Antibacterial and antifungal activity of a soda-lime glass containing copper nanoparticles. Nanotechnology.

[7] Fan C, Chu L, Rawls HR, Norling BK, Cardenas HL, Whang K (2011). Development of an antimicrobial resin--a pilot study. Dent Mater.

[8] Lipovsky A, Nitzan Y, Gedanken A, Lubart R (2011). Antifungal activity of ZnO nanoparticles--the role of ROS mediated cell injury. Nanotechnology.

[9] Paulo CS, Vidal M, Ferreira LS (2010). Antifungal nanoparticles and surfaces. Biomacromolecules.

[10] Kathiresan K, Alikunhi NM, Pathmanaban S, Nabikhan A, Kandasamy S (2010). Analysis of antimicrobial silver nanoparticles synthesized by coastal strains of *Escherichia coli and Aspergillus niger*. Can J Microbiol.

[11] Kim KJ, Sung WS, Moon SK, Choi JS, Kim JG, Lee DG (2008). Antifungal effect of silver nanoparticles on dermatophytes. J Microbiol Biotechnol.

[12] Panácek A, Kolár M, Vecerová R, Prucek R, Soukupová J, Krystof V (2009). Antifungal activity of silver nanoparticles against *Candida spp*. Biomaterials.

[13] Fesharaki PJ, Nazari P, Shakibaie M, Rezaie S, Banoee M, Abdollahi M (2010). Biosynthesis of selenium nanoparticles using *Klebsiella pneumoniae* and their recovery by a simple sterilization process. Braz J Microbiol.

[14] Shahverdi AR, Fakhimi A, Mosavat G, Fesharaki PJ, Rezaie S, Rezayat SM (2010). Antifungal Activity of Biogenic Selenium Nanoparticles. World Appl Sci J.

[15] Chen C, Koch LH, Dice JE, Dempsey KK, Moskowitz AB, Barnes-Eley ML (2010). A randomized, double-blind study comparing the efficacy of selenium sulfide shampoo 1% and ciclopirox shampoo 1% as adjunctive treatments for tinea capitis in children. Pediatr Dermatol.

[16] Zhang J, Wang X, Xu T (2007). Elemental selenium at nano size (Nano-Se) as a potential chemopreventive agent with reduced risk of selenium toxicity: comparison with se-methylselenocysteine in mice. Toxicol Sci.

[17] Jacobs MR, Bajaksouzian S, Peter C (2003). Appelbaum Telithromycin post-antibiotic and post-antibiotic sub-MIC effects for 10 Gram-positive cocci. J Antimicrob Chemother.

[18] Oremland RS, Herbel MJ, Switzer-Blum J, Langley S, Beveridge TJ, Ajayan PM (2004). Structural and spectral features of selenium nanospheres produced by Se-respiring bacteria. Appl Environ Microb.

[19] National Committee for Clinical Laboratory Standards (2002). Reference method for broth dilution antifungal susceptibility testing of filamentous fungi: Approved Standard M38-A.

[20] Abbass IM, Razak AA (1991). Cadmium, selenium, and tellurium chelators in *Aspergillus terreus*. Biol Trace Elem Res.

[21] Kulys J, Buch-Rasmussen T, Bechgaard K, Marcinkeviciene J, Christensen JB, Hansen HE (1993). Kinetics of glucose oxidase catalyzed electron transfer mediated by sulfur and selenium compounds. FEBS Lett.

